# Age-related changes in 'hub' neurons

**DOI:** 10.18632/aging.101606

**Published:** 2018-10-22

**Authors:** Abbi R. Hernandez, Sara N. Burke

**Affiliations:** 1 McKnight Brain Institute, Department of Neuroscience, University of Florida, Gainesville, FL 32610, USA; 2 Institute on Aging, University of Florida, Gainesville, FL 32610, USA

**Keywords:** cognition, glucose, ketone bodies, medial temporal lobe, metabolism, prefrontal cortex

Declining cognitive function is a hallmark of advanced age that can have profound effects on quality of life in older adults. Age-related changes in the central nervous system are first observed within the prefrontal cortex (PFC), which is responsible for executive functions and decision making, and the medial temporal lobe (MTL), which is critical for memory and stimulus recognition. Although the neurobiological mechanisms resulting in age-related cognitive impairments are not fully elucidated, it is clear that peripheral metabolic health and cognitive outcomes are reciprocally linked [1]. Among the most common consequences of aging is an inability to efficiently utilize glucose, resulting in declines in metabolic capacity. The PFC and MTL both show reduced glucose utilization [2] and are extensively reciprocally connected with each other as well as other brain structures. This anatomical organization requires that neurons in the PFC and MTL serve as neuronal hubs that connect disparate brain regions to enable cognition. Neuronal hubs are likely to be under higher metabolic demand and thus vulnerable to disruptions in declining metabolic capacity from altered glucose signaling. Two lines of evidence indirectly support this idea. First, humans [3] and animals [4] have disruptions in large-scale functional connectivity across the brain that correspond with cognitive decline in old age. Second, behaviors that require connectivity between PFC and MTL [5] are more sensitive at detecting age differences than behaviors that do not require communication between these structures [6].

Recently, a direct link between altered PFC activity patterns in aged rats and dysfunction in hub neurons has been shown by combining anatomical tracing with the imaging of cellular activity. While performing a PFC-MTL-dependent task, neuronal activity levels within these areas differed between young and aged rats, even when the aged rats were extensively trained to reach equivalent performance levels of the young [7]. Neuronal activity was quantified by labeling the mRNA products for *Arc*, an immediate-early gene transcribed in recently activated neurons. Interestingly, not only where age-related increases and decreases in activity significant within both of these regions, the age effects were specific to different cortical layers. When the activity levels were normalized to resting activity, the superficial layers of both the PFC and perirhinal cortex of the MTL had significantly fewer neurons positive for *Arc* in aged rats relative to young. Conversely, there was an age-related increase in neuronal activity within the deep layers of these regions.

**Figure 1:**
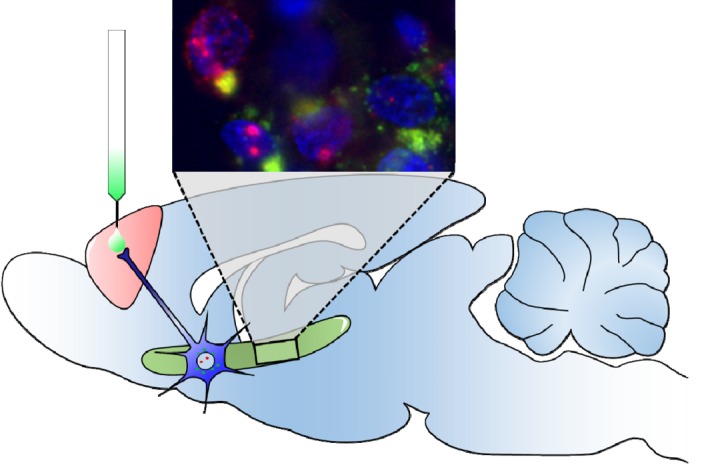
**Age-related alterations in neuronal activity patterns and hub neuron dysfunction were investigated in young and aged rats.** This was accomplished by combining anatomical tracing (retrograde tracer injected into the prefrontal cortex; green) and imaging neuron activity during behavior by labeling the immediate-early gene *Arc* (red). Thus, neurons (blue) with red and green signal are the perirhinal cortical projection cells to prefrontal cortex that were activated during behavior. Activity patterns significantly differed between aged and young rats, with changes primarily occurring within the hub neurons that have long-range projections across regions.

To determine if the age effects were specific to hub neurons connecting the PFC and perirhinal cortex, rats received an injection of fluorescently-conjugated Cholera Toxin-β (a non-toxic retrograde tracer) into the PFC (see Figure 1). This approach enabled the quantification of activity patterns in those neurons that project from the MTL to the PFC [7]. Interestingly, in all rats, the connected hub neurons were more likely to be active during behavior than the unconnected cells. Moreover, the age group differences were larger among the hub neurons than the cells that did not project to the PFC. These observations highlight the vulnerability of hub neurons to age-related dysfunction, suggesting a relationship with declining metabolic capacity in old age.

The function, morphology, and high activity levels of long-range projection neurons likely result in high metabolic demand. Therefore, when neurometabolism begins to decline with age, hub neurons comprising this interregional circuitry are the most vulnerable. This notion is corroborated by the observations that within the MTL there is an age-related decrease in the expression of glucose transporters [8] and prolonged decrease in extracellular glucose levels during behavior that does not quickly recover in aged animals [1].

If hub neurons are particularly vulnerability to declining glucose utilization and reduced metabolic capacity, this points to an intriguing therapeutic opportunity. In addition to glucose, neurons and many other cell types can utilize ketone bodies to provide ATP. Consumption of a ketogenic diet, which is being investigated as a metabolic intervention for the treatment of many neurological disease states [9], switches the body’s primary fuel source to ketone bodies. Unlike glucose transporters, ketone body transporter expression does not decline with age in the brain [8]. Moreover, we have recently demonstrated the ability of aged rats to enter a state of nutritional ketosis with positive changes in peripheral health, as well as the amelioration of age-related changes in other neuronal signaling-related proteins [8].

Together, these recent observations suggest that restoration of metabolic capacity may be able to produce beneficial effects on global brain organization. Ketogenic diets also reduce prevalence of diabetes and metabolic syndrome, both of which correlate with decreased cognitive outcomes and are the top two modifiable risk factors for neurodegenerative diseases.
